# Salbutamol-induced lactic acidosis in status asthmaticus survivor

**DOI:** 10.1186/s12890-021-01404-x

**Published:** 2021-01-12

**Authors:** Vorakamol Phoophiboon, Parima Singhagowinta, Sangdao Boonkaya, Thitiwat Sriprasart

**Affiliations:** 1 Division of Pulmonary and Critical Care Medicine, Department of Medicine, Faculty of Medicine, Chulalongkorn University, 1873 Rama 4 road, Pratumwan, Bangkok, 10330 Thailand; 2 Excellence Center for Critical Care Medicine, King Chulalongkorn Memorial Hospital, Thai Red Cross Society, Bangkok, Thailand; 3 Department of Medicine, Faculty of Medicine, Chulalongkorn University, Bangkok, Thailand

**Keywords:** Salbutamol-induced lactic acidosis, Status asthmaticus, Lactic acidosis, Salbutamol’s adverse effect, Case report

## Abstract

**Background:**

Salbutamol-induced lactic acidosis is a rare presentation that could manifest in specific clinical context as acute asthmatic attack treatment. An increase of glycolysis pathway leading to pyruvate escalation is the mechanism of hyperlactatemia in β2-adrenergic agonist drug.

**Case presentation:**

A 40-year-old man who had poor-controlled asthma, presented with progressive dyspnea with coryza symptom for 6 days. He was intubated and admitted into medical intensive care unit due to deteriorated respiratory symptom. Severe asthmatic attack was diagnosed and approximate 1.5 canisters of salbutamol inhaler was administrated within 24 h of admission. Initial severe acidosis consisted of acute respiratory acidosis from ventilation-perfusion mismatch and acute metabolic acidosis resulting from bronchospasm and hypoxia-related lactic acidosis, respectively. The lactate level was normalized in 6 h after hypoxemia and ventilation correction. Given the lactate level re-elevated into a peak of 4.6 mmol/L without signs of tissue hypoxia nor other possible etiologies, the salbutamol toxicity was suspected and the inhaler was discontinued that contributed to rapid lactate clearance. The patient was safely discharged on the 6th day of admission.

**Conclusion:**

The re-elevation of serum lactate in status asthmaticus patient who had been administrated with the vast amount of β2-adrenergic agonist should be considered for salbutamol-induced lactic acidosis and promptly discontinued especially when there were no common potentials.

## Background

Salbutamol-induced lactic acidosis is an unusual presentation. It could be diagnosed in patients who require the large amount of β2-adrenergic agonist in short period as status asthmaticus treatment. Although this salbutamol inhaler is a common drug rescuing airway obstructive problem, there has unexpectedly serious adverse effect—lactic acidosis when it is excessively applied. However, there have no definite diagnostic criteria, exclusion of other potential etiologies is essentially required. The pathophysiology of salbutamol-induced lactic acidosis is an increase of glycolysis pathway resulting in pyruvate and lactate escalation [1–5].

## Case presentation

A 40-year-old man was admitted to medical intensive care unit with acute hypercapnic respiratory failure due to status asthmaticus. His past medical history was poor-controlled asthma that intermittently used only a short-acting bronchodilator for 10 years. He was 12-pack-year smoker which quit over 10 years. He denied alcohol consumption nor recreational drug use. He presented with coryza, myalgia and low grade of fever for 6 days. On the day of admission, he developed difficulty of breathing, respiratory rate of 44 /minute. His blood pressure was 184/122 mm Hg and tachycardia of 120 /minute. An initial oxygen saturation was 85% at room air. On examination, he had poor air-entry with biphasic wheezing throughout the lung’s field. After intubation, he was deeply sedated and paralyzed in order to being controlled with a proper setting of mechanical ventilation. Arterial blood gas revealed acute respiratory acidosis with PH of 6.98, PaO_2_ of 90 mm Hg (under fraction of inspired oxygen-FiO_2_; 0.6) and PaCO_2_ of 119.5 mm Hg. The chest radiograph and initial serum investigations were unremarkable. Nasal swab for respiratory viral test was positive for Enterovirus**/**Rhinovirus. The essential investigations and dose of sedative drugs are shown in Table 1. During the admission, patient’s mean arterial pressure was over 65 mmHg without inotrope’s support. The salbutamol inhaler (100 mcg/puff) was administrated via ventilator’s inspiratory circuit, 4–8 puffs every 15–30 min. Furthermore, salmeterol/fluticasone propionate MDI (25 mcg*/*250 mcg) 4 inhalations every 12 h and four inhalations of tiotropium bromide soft mist inhaler (2.5 mcg) were used as inhaled controllers. Not only intravenous steroid was used for exacerbation regimen, but two grams of magnesium sulphate infusion was also given. Ceftriazone, azithromycin and oseltamivir were empirically started and discontinued when nasal swab, sputum culture and blood cultures revealed none of other co-infections. We did not use an aminophylline nor inhaled anesthetic agent due to unavailability. The ventilator setting was volume-controlled mode, 5 ml/kg of ideal body weight, 5 cm H_2_O of positive end expired pressure (PEEP), 0.6 of FiO_2_ and 60–100 L/min of decelerating flow. After 6 h of treatment, patient’s ventilation and oxygenation showed significant improvement contributing to lactate normalization (from 4.4 to 0.7 mmol/L). However, at 24 h of admission, his lactate level had re-elevated to a peak of 4.6 mmol/L contrary to the improvement of PaO_2_, PaCO_2_, patient’s symptoms and mechanical ventilator’s setting. There was a minimal rising of creatinine, potassium and creatinine phosphokinase (CPK) which was less likely to make a diagnosis of rhabdomyolysis. Furthermore, none of liver function test, urine analysis, lipid profiles and electrocardiogram’s finding had illustrated abnormality. Four and a half Litres of lactated Ringer’s solution were infusing for fluid resuscitation and maintenance during the first 48 h. The urine output throughout 48 h of admission was 45–180 ml/h with positive accumulative balance of 3.2 L. The salbutamol inhaler was discontinued while patient’s clinical status improved, a total dose of salbutamol was 31,500 mcg (315 puffs). The lactate level had rapidly decreased into normal range within 12 h of discontinuation and the patient was extubated safely at day 5 of the admission.

**Table 1:** Laboratory investigations, ventilator settings and sedative drugs

	0 h	6 h	12 h	24 h	32 h	48 h	54 h
**Arterial blood gas**							
PH	6.98	7.29	7.24	7.26	7.42	7.43	7.45
PaO_2_ (mm Hg)	90	91.9	119.2	95.6	61.9	79.2	76
PaCO_2_ (mm Hg)	119.5	55.6	48	52	44	42.8	38.4
HCO_3_ (mmol/L)	20	22	16	12	22	25	25
Serum lactate (mmol/L)	4.4	0.7	2.3	4.6	1.3	0.9	0.7
Creatinine phosphokinase (U/L)	–	–	1,877	2,007	–	1,454	880
Creatinine (mg/dL)	0.87	–	1.16	–	–	1.06	0.92
Potassium (mmol/L)	3.8	5.3	5.4	4.4	4	3.9	4.2
**Ventilator settings**							
Mode	VCV	VCV	VCV	VCV	VCV	PSV	PSV
Tidal volume (ml/kg)	5.5	5.5	4.3	4.3	6	6–7	6–7
Tidal volume (ml)	380–390	380–390	300	300	420	420–490	420–490
PEEP (cm H_2_O)	5	5	5	5	5	5	5
Respiratory rate (/min)	20–25	20–25	14	14	14	14	14
Flow (L/min)	60	60	70–100	70–100	60	–	–
FiO_2_	0.6	0.4	0.4	0.4	0.3	0.3	0.3
**Drugs**							
Propofol (mg/h)	–	200	100	100	60	off	–
Fentanyl (mcg/h)	50	80	50	80	80	80	50
Midazolam (mg/h)	4	5	6	6	4	off	–
Cisatracurium (mcg/kg/min)	–	2.2	2.2	off	–	–	–
Salbutamol inhaler (puffs)	N/A	N/A	N/A	Total 315 → off	–	–	–

*VCV* volume controlled ventilation, *PSV* pressure support ventilation, *PEEP* positive end-expiratory pressure

## Discussion and conclusion

Status asthmaticus is a severe stage of asthma exacerbation which required multimodalities of treatment such as high dose of bronchodilators, intubation with high mechanical ventilator’s setting and deep sedation. To effectively manage peripheral airway obstruction, salbutamol inhaler is a drug of choice for ameliorating bronchospasm but it remains uncertainty of maximum dose especially using through mechanical ventilator. The initial reduction of lactate level in the first 6 h as a result of improvement of hypoxemia and bronchospasm. In arterial blood gas analysis, the initial presentation was a combination of acute respiratory acidosis and metabolic acidosis which respiratory cause was a major contribution. When the bronchospasm had been improved, the overall PH was rising. However, the recovery of airway disease was not complete, in addition to the re-worsening of lactic acidosis predisposing to ongoing acidosis. Our case demonstrated an explicit point of lactate level’s rebound after normalization, there were none of other potential etiologies of lactate’s re-elevation such as persistent hypotension, uncontrolled viral infection, hospital-acquired infection, abnormal lactate clearance relating to liver or renal dysfunction. The medication-induced lactatemia as type B (non-hypoperfusion cause) should be considered in this case which is reported in several mechanisms of lactate production [5–7] However, the potential drugs in our case were salbutamol inhaler and propofol infusion, which propofol is unlikely presented without propofol infusion syndrome (PRIS). PRIS generally consists of rhadomyolysis, abnormal lipid profiles and cardiac dysfunction, the uncoupling oxidative phosphorylation is the mechanism of PRIS-related hyperlactatemia [8–10]. Furthermore, lactated Ringer’s solution does not seem to increase circulating lactate concentration unless infusing large volumes (180 ml/kg/h) [11, 12].

Given there are no diagnostic criteria of salbutamol-induced lactic acidosis, drug levels nor determined dose, deliberate clinical evaluation and exclusion are essential in this context. There was a retrospective cohort that reported the 1200 mcg of albuterol causing lactic acidosis in a couple hours whereas our case was used in the higher amount [13]. As a systematic review, the lactate level of drug-induced hyperlactatemia was reported in wide range and 1.9 days was a median time of lactate clearance [4]. In our case illustrated the suspected salbutamol-induced lactic acidosis that showed the using dose (31,500 mcg), the duration of onset (within 24 h), the peak of drug-induced lactate (4.6 mmol/L) and the clearance of drug-induced lactate (8 h). This observational information may be useful for guiding diagnosis. After salbutamol inhaler discontinuation, the lactate level decreased rapidly, so salbutamol-induced lactic acidosis was diagnosed. Although there are some mechanisms reported that glucocorticoid enhances the efficacy of β2-adrenergic agent which may or may not worsen lactic acidosis, the benefit of glucocorticoid in asthmatic attack treatment is obvious [14]. For further study, the implementation of either drug toxicity level or minimum accumulative amount to confirm the diagnosis is essentially required.

Salbutamol-induced lactic acidosis is a rare presentation, potentially occurred when severe asthmatic attack diagnosed. The increase of glycolysis and pyruvate production without poor perfusion state is the main mechanism of lactate production in β2-adrenergic agents. Exclusion of other potential etiologies of lactic acidosis is necessary, but salbutamol-induced lactic acidosis should also be aware in this context.

## Data Availability

All data are available in the manuscript [and its supplementary information files].

## Abbreviations

VCV: Volume controlled ventilation
PSV: Pressure support ventilation
PEEP: Positive end-expiratory pressure
FiO_2_: Fraction of inspired oxygen
CPK: Creatinine phosphokinase
PRIS: Propofol infusion syndrome
